# Clinically significant changes in genes and variants associated with epilepsy over time: implications for re-analysis

**DOI:** 10.1038/s41598-024-57976-1

**Published:** 2024-04-02

**Authors:** Alan J. Robertson, Khoa A. Tran, Carmen Bennett, Clair Sullivan, Zornitza Stark, Lata Vadlamudi, Nicola Waddell

**Affiliations:** 1 Faculty of Medicine, University of Queensland, Brisbane, Australia; 2 Medical Genomics Group, QIMR Berghofer Medical Research Institute, Brisbane, Australia; 3 Queensland Digital Health Centre, University of Queensland, Brisbane, Australia; 4 The Genomic Institute, Department of Health, Queensland Government, Brisbane, Australia; 5 School of Biomedical Sciences, Queensland University of Technology (QUT), Brisbane, Australia; 6 UQ Centre for Clinical Research, Herston, Brisbane, QLD 4029 Australia; 7 Department of Neurology, Royal Brisbane & Women’s Hospital, Herston, Brisbane, QLD 4029 Australia; 8 Centre for Health Services Research, Faculty of Medicine, The University of Queensland, Woolloongabba, Australia; 9 Department of Health, Metro North Hospital and Health Service, Queensland Government, Brisbane, Australia; 10 Victorian Clinical Genetics Services, Murdoch Children’s Research Institute, Melbourne, VIC Australia; 11 Australian Genomics, Melbourne, Australia; 12 University of Melbourne, Melbourne, Australia

**Keywords:** Re-analysis of genomic data, Epilepsy, Genetic testing, PanelApp, PanelApp Australia, ClinVar, Genome data integration, Genetics, Molecular medicine

## Abstract

Despite the significant advances in understanding the genetic architecture of epilepsy, many patients do not receive a molecular diagnosis after genomic testing. Re-analysing existing genomic data has emerged as a potent method to increase diagnostic yields—providing the benefits of genomic-enabled medicine to more individuals afflicted with a range of different conditions. The primary drivers for these new diagnoses are the discovery of novel gene-disease and variants-disease relationships; however, most decisions to trigger re-analysis are based on the passage of time rather than the accumulation of new knowledge. To explore how our understanding of a specific condition changes and how this impacts re-analysis of genomic data from epilepsy patients, we developed Vigelint. This approach combines the information from PanelApp and ClinVar to characterise how the clinically relevant genes and causative variants available to laboratories change over time, and this approach to five clinical-grade epilepsy panels. Applying the Vigelint pipeline to these panels revealed highly variable patterns in new, clinically relevant knowledge becoming publicly available. This variability indicates that a more dynamic approach to re-analysis may benefit the diagnosis and treatment of epilepsy patients. Moreover, this work suggests that Vigelint can provide empirical data to guide more nuanced, condition-specific approaches to re-analysis.

## Introduction

Epilepsy is a common and potentially debilitating group of neurological conditions, with a broad spectrum of phenotypes^1,2^. While there are a range of known causes for epilepsy including infection, trauma, and stroke^1,3–5^; advances in genomics have shown that for more than two thirds of epilepsy patients, where the cause is unknown, genetic factors are likely to play a role^1,6–9^. As a molecular diagnosis can provide patients with tailored therapeutic interventions, detailed information about the patient’s prognosis and more accurate genetic counselling^10–12^, genetic testing is increasingly being offered to patients with epilepsy, without a known cause^2,9,13^.

The genetic drivers of epilepsy are complex^14^. Only a small fraction of patients with inherited epilepsy are believed to have a Mendelian form of the disease; with the remaining proportion having complex, polygenic drivers of their condition^14^. Even in the groups of epilepsy patients that typically have higher diagnostic yields when tested, many individuals do not receive a molecular diagnosis after genomic testing^15,16^.

Re-analysing existing genomic information has emerged as a method to provide molecular diagnoses to patients with previously uninformative genomic testing results^17–20^. Meta-analyses of existing studies indicate that re-analysing genomic data after 24–36 months increases the diagnostic yield by 10–15%^18–20^. A number of mechanisms are responsible^19^, chief amongst them being the discovery of new gene-disease and variant-disease associations, which account for 62.5% of new diagnoses reported in the literature^18^.

As different diseases involve different numbers of genes, and are subject to differing levels of research and clinical testing, the rates of discovery of (and refinement of existing) gene-disease associations are not uniform. As a result, our understanding of the genetic component of some conditions is changing faster than others, indicating that patients with different conditions could benefit from different re-analysis intervals^21–23^. However, as the majority of re-analysis studies examined large heterogeneous groups of patients^19,20^, it has been difficult to investigate this further.

In a recent study, we characterised the different ways the genes used to diagnose different conditions has changed, in order to explore how our understandings of the molecular components of different conditions are changing^21^. We characterised how the genes in 113 virtual gene panels in PanelApp Australia changed over a period of 2.5 years. The Genetic Epilepsy panel was found to be accumulating new diagnostic grade genes significantly faster than the majority of other panels^21^. As epilepsy is one of the few conditions, in which the clinical potential for re-analysis-enabled care in has been established^15,16,24^, and given our previously study only included a single epilepsy gene panel and did not examine how the variant-disease associations changed over time, we elected to explore this condition.

We developed Vigelint (Variants in Genes Linking Tool), a novel, largely automated bioinformatics approach that combines gene and variant information to characterise how our understanding of the molecular components of a specific disease changes over time. Vigelint incorporates and upgrades our previous tool, PADA-WAN, allowing us to track the evolution of five virtual epilepsy gene panels over a period of five years from PanelApp Australia and Genomics England PanelApp. Vigelint also includes two new tools that are used to integrate the genes in a panel with the corresponding variant information from ClinVar. The results from Vigelint provide new insights for determining when to re-analyse genomic data from epilepsy patients and, represent an approach that can be used to explore how the molecular information related to other conditions changes.

## Methods

### Study Design—selecting PanelApp as means to study how clinically significant epilepsy genes change over time

PanelApp Australia^25^, and the Genomics England instance of PanelApp^26^ were selected to provide the epilepsy panels for this analysis. The PanelApp platform provides a centralised curation system to collate a list of genes that should be included on each panel^26^. The platform is open to a wide range of contributors, while a curation team critically assess evidence and determines final gene inclusions and ratings. The panels from PanelApp are freely-available, and every change (and the supporting rationale) is openly recorded and accessible, which means it is possible to determine the precise ways a panel changes over time^26^. Two instances of PanelApp (United Kingdom and Australia) are available, each of which maintains a separate suite of panels, and are used in clinical settings^25,26^.

Other ‘open’ resources, such as Genes4Epilespy^27^ were considered for this analysis. As the number of clinically important genes in a panel can rapidly change, we wanted to ensure this work could identify similar changes, however these alternative gene lists did not support the same monthly resolution offered by PanelApp. As a result, we focused this work to the panels developed and maintained by PanelApp and PanelApp Australia.

### Defining the vigelint pipeline

In order to examine how our understanding of the clinically-significant variants, in clinically-significant genes changed over time, we developed *Vigelint*. This pipeline is made up of three separate tools. PanelApp Downloader and Analyser—Web Application Navigator (PADA-WAN) was designed to download every version of a panel from PanelApp Australia, and determine how the specific genes used test a condition change overtime. PADA-WAN was originally described in a prior publication^21^, but was expanded to support both the Genomics England instance of PanelApp. ClinVar Period Organiser (CVPO), was developed for the Vigelint pipeline. CVPO takes the official, monthly Gene Specific Summary files from ClinVar, and combines all the data into a querible matrix, making it possible to observe how the number of variants within each gene changes each month. Another tool written for Vigelint is Join External genetic Disease Information (JEDI). JEDI was written to characterise how the clinically significant information associated with a condition changes over time. By combining the variant information from CVPO with the gene information from PADA-WAN, JEDI makes it possible to determine the number of clinically significant variants in clinically significant genes at any given point in time.

Vigelint is available at: github.com/MedicalGenomicsLab/Vigelint/

The results presented here exclusively focus on epilepsy. However, Vigelint was written with the intent to combine the information from any PanelApp panel with the results from ClinVar. While a user will be required to rename the Gene Specific Summary files and ensure that the settings in the parameters file are correct, Vigelint is a largely automated process and will collect, process and combine this data with very little user input.

### Study design—identifying the gene panels that examine epilepsy

Every panel present within Genomics England PanelApp^26^ and PanelApp Australia^25^ was examined to identify panels that could potentially be associated with epilepsy. These potential panels were reviewed, and those that could be confidently associated with epilepsy were selected for further analysis (A.R. and Z.S.). The panel identifier for each selected panel were recorded. The age of the epilepsy panels within these databases were determined, and the oldest was used to define the date range of the ClinVar analysis.

### Capturing and summarising the information from genomics England PanelApp and PanelApp Australia

PADA-WAN was used to download every version of the selected epilepsy panels from PanelApp and PanelApp Australia between April 4th 2018 to April 30th 2023. The changes in each panel were characterised for each month over the analysis window. The version of the panel present on the last day of the month at 11:59 pm was selected as the panel that represents that month.

### Creation of the epilepsy gene list

To produce a list of genes known to be associated with epilepsy, we extracted the genes from each of selected epilepsy panels from Genomics England PanelApp and PanelApp Australia and combined them into a single list (Supplementary Table 2). A gene was classified as a diagnostic gene, if it was classified as diagnostic (corresponding to Green status on PanelApp) in at least one of the epilepsy panels on April 30^th^ 2023.

### Retrieving variant information from ClinVar

To determine the number of variants that aligned to each gene, we utilised the information present in the official the Gene Specific Summary file from ClinVar. For each month between April 2018 to May 2023, the tab delimited Gene Specific Summary file was downloaded from the ClinVar FTP server^28^. A shell script to download these files is available as part of CVPO. As ClinVar does not apply dates in the same manner as PanelApp, each of these ClinVar files were manually re-named with the closest corresponding timepoint from PanelApp (Supplementary Table 1).

### Combining the variant information to produce the variant matrix

CVPO extracts data from the ‘total alleles’, and the ‘alleles reported to be Pathogenic and Likely Pathogenic (P/LP)’ columns from the each of the monthly ClinVar Gene Specific Summary files, and combines all this information into an organised matrix. This matrix is queryable, making it possible to look up the number of variants known to be associated with each gene for each month of the analysis window. It should be noted that while the Gene Specific Summary files uses the term, ‘allele’ to refer all types of alleles, in the ‘Total_alleles’ column, the term ‘alleles’ in ‘Alleles_reported_Pathogenic_Likely_pathogenic’ excludes variants that overlap other genes.

### Annotating the variant matrix with an alternative Gene ID to facilitate matching

To look up the number of variants that align to a gene, from a PanelApp panel, each gene from in the CVPO matrix was annotated with an Ensembl Gene ID, as PanelApp describes genes using the Ensembl annotation of the genome, and ClinVar uses NCBI Gene IDs to store variant information. To achieve this the Ensembl BioMart web application was used to create an up-to-date list that contained the NCBI ID and the corresponding Ensembl Gene ID for the same loci. A script to programmatically generate this file, is available as part of CVPO. The information in this file was ingested by CVPO and used to annotate each loci.

In the instances when a gene in one database, would match to multiple genes in the other, the Ensembl and NCBI gene models were reviewed, and the gene that contained the largest number of variants was selected as the representative gene for the loci. Some genes were only present in one database, and would be labelled as ‘NA’.

Preliminary work revealed that some of the epilepsy genes had an Ensembl Gene ID, that did not correspond to an NCBI Gene ID. To address this and ensure that the ClinVar was properly utilised, each epilepsy gene that was not associated with an NCBI Gene ID was manually reviewed, and compared to resources such as the HUGO HGNC symbol checker^29^, Uniprot^30^ and Gene Cards^31^ to identify potential matches that weren’t present in the file obtained from the Ensembl Biomart. The Epilepsy Gene List was updated to reflect this alternative identifiers.

### Joining variant information and specific genes

JEDI (Join External genetic Disease Information) is a series of scripts (Python and R) designed to combine gene information with variant information, and examine how this information changes over time. As gene information can either be static or changing, two different versions of JEDI have been developed, *Static Gene List Mode* and *Evolving Gene Panel Mode*.

### Combining the information in PanelApp and ClinVar–static gene list

Static Gene List Mode, was used to extract each gene present in the *Epilepsy Gene List,* (Supplementary Table 2), identify the NCBI ID and use this information to determine corresponding number of variants in the large ClinVar matrix that had been produced by CVPO. This approach made it possible to annotate each of the epilepsy genes with the number of variants that aligned to that gene for each month between April 2018 and April 2023. This approach also provided the number of P/LP variants associated with the gene.

### Combining the information in PanelApp and ClinVar—evolving panel

The Evolving Gene Panel Mode combines the results from PanelApp and ClinVar for a specific time point, for each month during the analysis window. This approach approximates the information available to curators at a specific point in time. This is achieved by determining the specific version of a panel present at a point in time, opening the appropriate version of the panel and for each gene within, identifying the number of variants known to lie within that gene from ClinVar at the corresponding time point. The JEDI script was run for each of the epilepsy panels.

### Determining changes in the number variants in the ClinVar data

The change in the number of variants between time points was calculated as a percentage increase. When the individual variants in *GFAP* were characterised to explore how, this was performed by downloading the variant summary files from ClinVar, and using the grep command in MacOS to extract the records that aligned to the *GFAP*.

### Ethical approval

The information retrieved from PanelApp, PanelApp Australia and ClinVar does not contain any patient data, and as a result, does not require ethics approval.

## Results

### Analysis of virtual gene panels shows that the number of diagnostic genes associated with epilepsy is increasing

Between Genomics England PanelApp, and PanelApp Australia, five virtual gene panels associated with epilepsy met the criteria for analysis (Table 1). The panels were classified as either; Specific panels (n = 3); smaller, targeted panels used to examine the genes associated with specific forms of epilepsy or Broad panels (n = 2), larger panels designed to examine the broader clinical indication.

**Table 1:** Summary of the panels related to epilepsy from PanelApp and PanelApp Australia.

Panel name	Source	Panel type	Initial release date	2023–04-30 version	Total number of iterations	Total No. genes*	Total No. diagnostic grade genes**
Early onset or syndromic epilepsy (EOSE)	Genomics England PanelApp	Broad	April 2018	4.18	2834	792	521
Genetic epilepsy	PanelApp Australia	Broad	November 2019	0.1843	1842	823	668
Progressive myoclonic epilepsy	PanelApp Australia	Specific	January 2020	0.16	17	33	31
Familial generalised epilepsy	PanelApp Australia	Specific	November 2021	0.13	14	20	15
Focal epilepsy	PanelApp Australia	Specific	November 2021	0.13	14	16	13

*All values reported here describe each of the Gene Panels as of the 30th of April 2023.

**PanelApp Panels include genes, short tandem repeats and regions commonly impacted copy number variations, here we exclusively focus on the genes.

**Figure 1:**
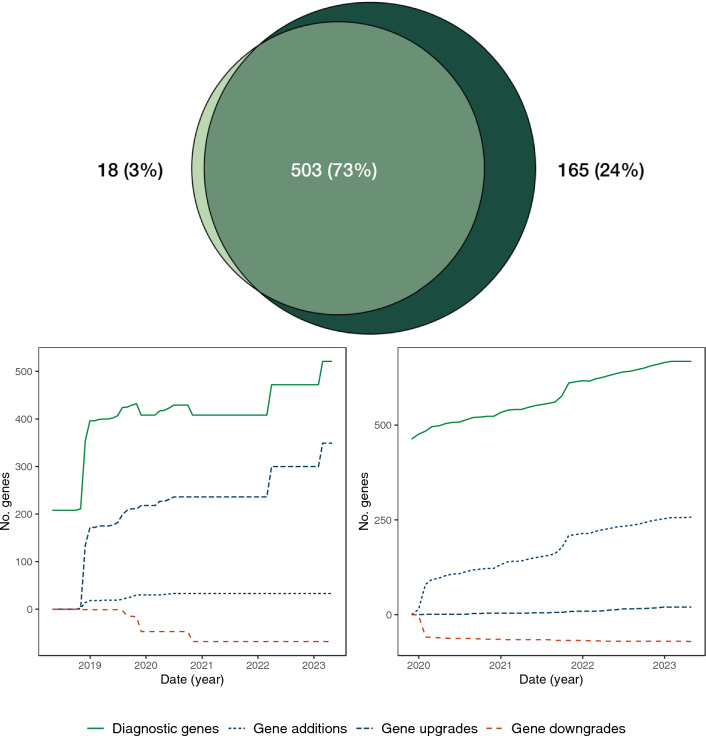
An overview of the Epilepsy Gene Panels from PanelApp and PanelApp Australia. (**A**) The overlap in the diagnostic genes that make up the EOSE and Genetic Epilepsy panels. the number of diagnostic genes on the EOSE panel is shown in light green, and the number diagnostic genes from the Genetic Epilepsy panel is shown in dark green. (**B**) Gene Changes in the broad epilepsy panels over time. The number of diagnostic genes in each panel for each month of the analysis window (x-axis) is shown in green. The different types of shown gene changes are also shown (y-axis). The Gene Changes that increase the number of diagnostic genes are shown in blue, while those that reduce the number are shown in red. As only a single gene was removed from these panels (Genetic Epilepsy panel) this information is not displayed on this graph.

The two broad panels contained more genes than the specific panels, had received the largest number of iterations, and had existed for the longest amounts of time (Table 1). The Genomics England PanelApp Early Onset or Syndromic Epilepsy (EOSE) panel was previously known as the ‘Genetic Epilepsy Syndromes’ panel, and is used by the NHS Genomic Medicine Service^25^. The PanelApp Australia Genetic Epilepsy panel is designed for the same clinical indication. A comparison of the April 2023 versions of each panel revealed that 503 diagnostic genes were present in both (Fig. 1A). The EOSE panel contained 18 unique genes, and the Genetic Epilepsy panel had 165, highlighting the differences in testing available between different healthcare settings.

To determine how the genes associated with the broad epilepsy panels have changed over time we examined the individual genes that were present on the EOSE and the Genetic Epilepsy panels at the end of each month since the initial release of each panel up until April 2023. When released in April 2018 the EOSE panel contained 521 genes, of which 208 were classified as diagnostic. By April 2023, the total number of genes in the panel had increased to 792, of which 521 were diagnostic genes. Examining the individual Gene Changes (Fig. 1B), defined as the number of new diagnostic genes or genes moving from diagnostic to non-diagnostic status, revealed that 33 new diagnostic genes were added to the panel, while a further 349 genes were upgraded to diagnostic status. Sixty-eight genes were downgraded from diagnostic status. These changes did not occur at a constant rate. Of the 382 diagnostic genes gained by the panel during the analysis window, 187 occurred between October and December 2018 (Fig. 1B). Conversely there were also long periods in which the number of diagnostic genes did not change (Fig. 1B).

Between the launch of the PanelApp Australia Genetic Epilepsy panel in November 2019 and April 2023, the total number of genes in the panel increased from 463 to 823, and the number of diagnostic genes increased by 205 to 668. Examining the Gene Changes over the analysis window revealed the addition of 257 new diagnostic genes, and removal of a single diagnostic gene, with a further 20 genes upgraded to diagnostic status and 71 genes downgraded from diagnostic status (Fig. 1C). While the diagnostic genes on the Genetic Epilepsy panel changed at more constant rate than the EOSE panel (Fig. 1C), this panel still saw ‘bursts’ of new diagnostic genes. For example, in October 2021, the total number of diagnostic genes available to laboratories increased by more than 5%.

The Specific gene panels did not change in the same ways as the Broad panels. The panels designed to examine the genetic components of Focal Epilepsy and Familial Generalised Epilepsy did not see any Gene Changes during the analysis period. However, the Progressive Myoclonic Epilepsy panel did change. Between January 2020 and April 2023, one gene was downgraded (Feb 2020) and one gene was upgraded to diagnostic grade (April 2022).

### Analysis of ClinVar show different ways the number of clinically significant variants in a cohort of epilepsy genes change

Resources that collate variant information, such as ClinVar, play an important role in re-assessing the pathogenicity of a variant^19^. To examine how the evidence available to curators evolves, changes in the total number of alleles, and the number of P/LP variants in ClinVar were examined. To achieve this, each month the number of variants in ClinVar were determined. We restricted the analysis to the variants, within the 668 genes which were classified as diagnostic on at least one of the PanelApp epilepsy panels (Supplementary Table 2), to focus on the variants most likely to produce new diagnoses for epilepsy patients.

**Figure 2:**
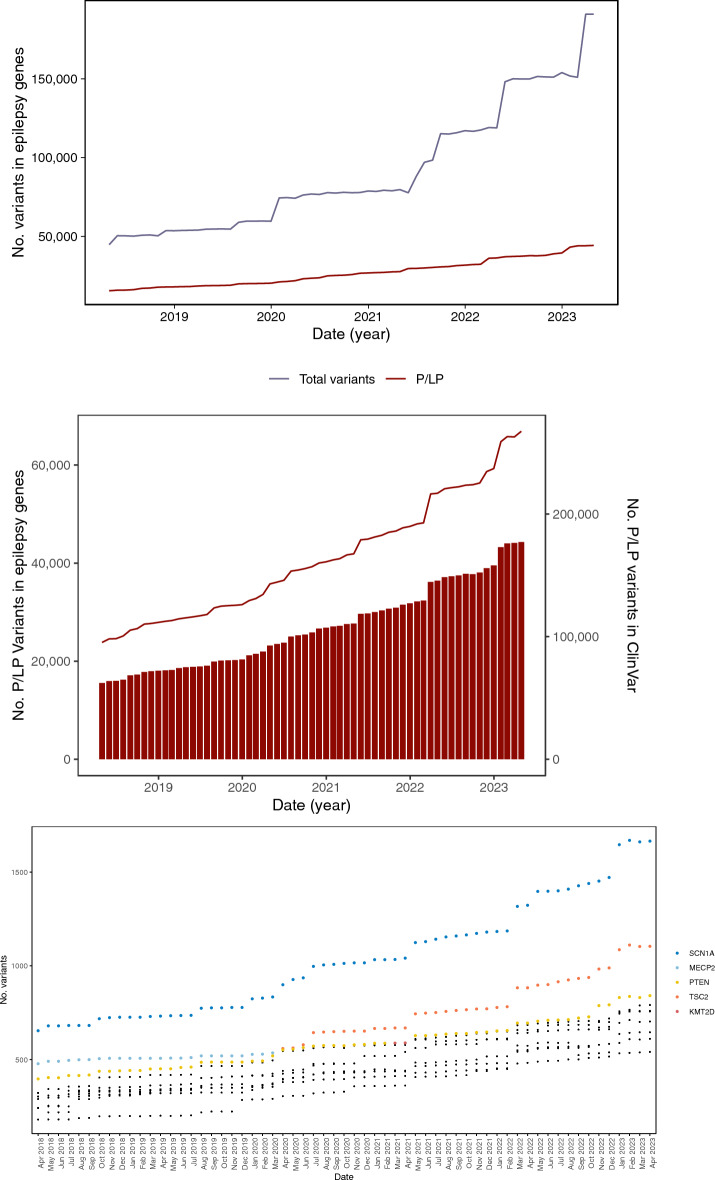
The number of variants in diagnostic epilepsy genes between 2018 and 2023. (**A**) The number of variants in the 668 epilepsy genes for each month between April 2018 and April 2023. The total number of alleles is shown in lilac, with the relative proportion of these variants classified as P/LP shown in maroon. (**B**) Changes in the number of P/LP variants. The maroon bars show the number of P/LP variants in the 668 epilepsy genes. The maroon line uses the secondary y-axis and represents the total number of P/LP variants within all of ClinVar for each month of the analysis window. (**C**) The number of P/LP variants in the genes that contain the greatest number of variants. The three genes with the largest number of P/LP variants each month are highlighted as a coloured point, with the respective gene name shown in a matching colour.

In April 2018, there were a total of 80,174 variants across the 668 diagnostic epilepsy genes (Supplementary Table 3). By April 2023, the total number of variants in these genes had increased to 374,057 (466%, Fig. 2A). Examining the number of variants within the epilepsy genes in monthly snapshots, showed periods when the total number of variants rapidly increased.

Between April 2018 and April 2023, the number of P/LP variants within the 668 diagnostic genes almost tripled from 15,542 to 44,298 (Fig. 2B). The number of P/LP variants in the epilepsy genes increased by an average of 1.8% per month (Supplementary Table 4), however, there were multiple months which saw large increases (> 5.0% per month). For example, between February and March 2022, the number of P/LP variants in these genes increased by 11.8%. High levels of correlation between the number of P/LP variants in the epilepsy genes, and all the P/LP variants listed in ClinVar were detected (Supplementary Table 5). Similarly, comparing the monthly changes in P/LP variants between the epilepsy genes all the entirely of ClinVar revealed a similar trend (Fig. 2B).

Further restricting our analysis to *individual* diagnostic genes showed that on average, the number of P/LP variants within each epilepsy gene increased by 42 P/LP variants (median = 16, range −52 to 1011, Supplementary Table 6) between April 2018 and April 2023. There was no significant correlation between the changes in the number of P/LP variants in a gene and the length of the gene (Pearson = 0.08). The genes associated with the largest increases over the entire analysis window were *SCNA1* (+ 1011), *TSC2* (+ 782) and *LAMA2* (+ 635). Curiously, the number of variants in 18 of the expert reviewed, diagnostic grade genes, did not change, while the number of P/LP variants decreased in an additional 14 genes (Supplementary Table 5). Detailed examination of *GFAP*, the gene with the largest decrease of P/LP variants, revealed that 5 variants had moved from pathogenic to conflicting status, 4 had been re-classified as VUS and 58 were now listed as ‘Not Provided’. Further investigation of the variants listed as ‘Not Provided’ suggested that previous evaluations had been removed from ClinVar.

To study this data at a more granular level, the number of the number of P/LP variants in each diagnostic epilepsy gene was examined for each month of analysis window (Fig. 2C). This work revealed that 10 genes contained ~ 20% of the total number of P/LP variants in any given month (Fig. 2C). For every month in the analysis window, *SCN1A* contained the greatest number of P/LP variants (April 2018 = 654, April 2023 = 1655). This is unsurprising, given *SCN1A’s* well documented role in epilepsy^32^. The position of other genes in this list were not as stable.

### Combining gene-disease and variant-disease associations for each month of the analysis window to determine the amount diagnostic information available to laboratories

To concentrate on the changes that are most likely to produce new diagnoses, we determine how the number of P/LP variants associated with each of the epilepsy panels changed over time. To achieve this, we extracted individual diagnostic genes that were present at the end of each month, for each panel, and determined the number of P/LP variants that were present in ClinVar at the same time point. This approach allowed us to track how the clinically significant information available to laboratories using these panels changed for each month between 2018 and 2023.

**Figure 3:**
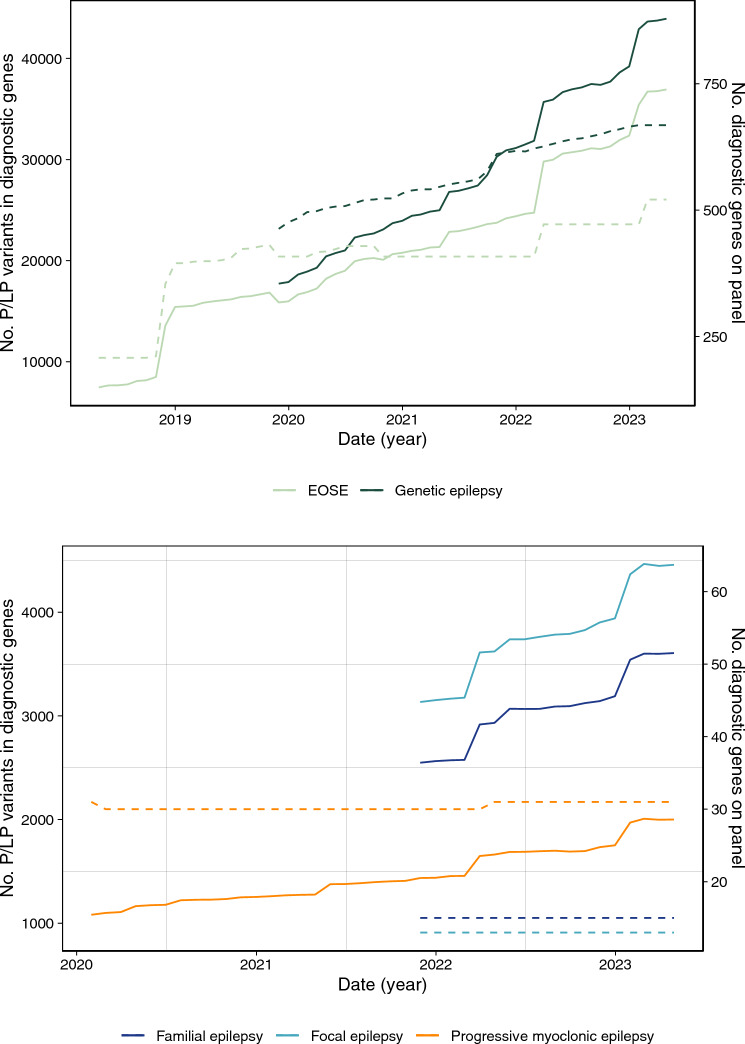
the number of P/LP variants in diagnostic genes for each month between April 2018 and April 2023. (**A**) Changes in the Broad panels between April 2018 and April 2023 (x-axis). The number of P/LP variants in diagnostic genes (thick lines) is shown on the left y-axis, while the number of diagnostic genes (thin lines) in each panel is shown in the right y-axis. The EOSE panel is shown in dark green, the Genetic Epilepsy panel in bright green. (**B**) Changes in the Specific panels between April 2018 and April 2023 (x-axis). The number of P/LP variants in diagnostic genes (thick lines) is shown on the left y-axis, while the number of diagnostic genes (thin lines) in each panel is shown in the right y-axis. The Progressive Myoclonic Epilepsy Panel is shown in orange, the Familial Epilepsy Panel is dark blue, and the Focal Epilepsy Panel is light blue.

On average, the number of P/LP variants in diagnostic genes increased by 3.0% each month in the EOSE panel (median = 1.0%, range = − 5.8 to 59.6%) and 2.3% in the Genetic Epilepsy Panel (median = 1.2%, range = − 0.2 to 12.1%) (Table 2). The EOSE panel saw the most change during the analysis window (394% increase in the number of P/LP variants in available diagnostic genes), while the Genetic Epilepsy panel consistently provided laboratories with a larger number of P/LP variants in diagnostic genes, reflective of the larger panel size (Fig. 3A). When examining the changes in EOSE panel for the same period as the Genetic Epilepsy panel, (November 2019 to April 2023) the number of diagnostic genes grew by 113 (28%) and the P/LP variants in those genes increased by 21,058 (133%). The month-to-month changes for both Broad panels revealed periods of high increase in the number of available diagnostic P/LP variants (Fig. 3A).

**Table 2:** The evolution of each panel from launch until April 2023.

Panel name	Analysis window	Versions compared*	Total No. genes**	No. variants in all genes	No. diagnostic genes**	No. P/LP variants in diagnostic genes	Increase in diagnostic genes***	Increase in PLP in diagnostic genes**
EOSE genomics england ID: 402 class: BROAD	60 months	0.507 (2018–04-30)	519 (521)	75,551	208 (208)	7477	**313** (150%)	**29,453** (394%)
		4.18	790 (792)	419,670	521 (521)	36,930		
Genetic Epilepsy PANELAPP AUSTRALIA ID: 202 class: BROAD	41 months	0.14 (2019–11-30)	463 (464)	100,624	462 (463)	17,726	**205** (44%)	**26,206** (148%)
		0.1843	822 (823)	423,954	667 (668)	43,932		
Progressive Myoclonic Epilepsy PANELAPP AUSTRALIA ID: 331 class: SPECIFIC	39 months	0.1 (2020–01-31)	31 (31)	7,803	31 (31)	1081	**0** (0%)	**920** (85%)
		0.15	33 (33)	19,403	31 (31)	2001		
Familial Generalised Epilepsy PANELAPP AUSTRALIA ID: 3795 class: SPECIFIC	17 months	0.12 (2021–11-30)	20 (20)	14,978	15 (15)	2549	**0** (0%)	**1057** (41%)
		0.13	20 (20)	22,890	15 (15)	3606		
Focal Epilepsy PANELAPP AUSTRALIA ID: 3796 class: SPECIFIC	17 months	0.1 (2021–11-30)	16 (16)	21,924	13 (13)	3134	**0** (0%)	**1324** (42%)
		0.13	16 (16)	33,033	13 (13)	4458		

*We compare the version of a panel from April 30th, 2023, to the Launch Window version of a panel. The Launch Window version of a panel is the version present on the last day of the month the panel was released in.

**It is not always possible to match an Ensembl ID (used by PanelApp) to an NCBI ID (used by ClinVar) the number of matching genes (above) is reported separately to the total number of genes in the panel (below). Subsequent numbers are the result of the matching genes.

***The increase is shown as the number of diagnostic genes (above), and the increase as a proportion of the number of diagnostic genes in the launch window version.

****The increase is shown as the number of P/LP variants (above), and the increase as a proportion of the number of P/LP variants the launch window version.

Significant values are in bold.

The largest increase detected was in the EOSE panel between October and November 2018, where the number of diagnostic variants increased by 59.6%. This can be explained by the number of EOSE diagnostic genes increasing by 67.8% (n = 143) in the same period. An increase in March 2022 was present in both Broad panels. The number of diagnostic P/LP variants in the EOSE panel increased by 20.5%, with the number of diagnostic genes in the EOSE panel growing by 15.7% (n = 64). The number of P/LP variants in the Genetic Epilepsy panel also grew by 12.1% during March 2022, however the number of diagnostic genes in the Genetic Epilepsy panel only increased by 0.6% (n = 4). Examining ClinVar, revealed that the total number of P/LP variants increased by 10.9% during the same period, indicating that observing the changes in genes, and the changes in variants are needed to understand how the publicly available data about a condition is evolving.

In the Specific panels, despite low amounts of gene changes, the number of P/LP variants still increased (Table 2). The number of P/LP variants associated with the Familial Generalised Epilepsy Panel and the Focal Epilepsy panels increased by 41–42%. Whereas for the Progressive Myoclonic Epilepsy panel, which is an older panel, the number of available P/LP variants increased by 85%. In addition to observing large increases in the number of available variants (Fig. 3B), examining how the amount of available information changes each month of the analysis window for the Specific panels, also revealed that even when the diagnostic genes associated with a panel remain stable, clinically significant changes still occur.

## Discussion

In a previous study, we characterised how a subset of panels within PanelApp Australia evolved over a period of two years and a half-years^21^. This work demonstrated that the Genetic Epilepsy panel had one of the largest increases in diagnostic genes (Genetic Epilepsy panel 244, vs. median broad panel = 27). From these results, we suggested that individuals who did not receive a molecular diagnosis after testing would be likely to benefit from re-analysis intervals shorter than the typical 24–36 months. However, the new variant information is another key driver for new diagnoses, and this study did not examine how the P/LP variants in these genes changed or how similar panels from other healthcare systems changed over time. The work presented here builds on this foundation, examines panel evolution from two healthcare settings at a much higher resolution and shows how the combined diagnostic information (genes and variants) has changed.

Changes in the relationship between a specific condition and the genes/variants associated with that condition were identified as the primary drivers of new diagnoses by studies of the re-analysis literature^18,19^. Be it through the discovery of new relationships, the refinement of existing relationships or being able to identify relevant relationships at the time of curation, the ways these relationships change are clinically significant^19^. By examining how the genes associated with each epilepsy panel from Genomics England PanelApp and PanelApp Australia changed overtime and characterising how the variants associated within each of these genes have evolved, we were able to determine how our understanding of the molecular components of epilepsy has changed over a period of five years.

The results presented here describe different ways in which our understanding of the genes and variants associated with epilepsy has changed and is incorporated into public databases. There were periods that saw relatively steady increases in the number of clinically significant P/LP variants available to laboratories. However, there were also periods when the number of diagnostic genes and P/LP variants rapidly increased. These periods were shown to increase the amount of P/LP variants in diagnostic genes by as much as 59.6% in a single month. Understanding these patterns will impact on when and how genomic data from epilepsy patients should be re-analysed.

As we previously suggested that patients tested with the Genetic Epilepsy panel would benefit from a shorter re-analysis interval due to the large number of diagnostic genes changes^21^, we also examined the Genomics England PanelApp EOSE panel to see if these patients would benefit from the same recommendation. While the PanelApp Australia Genetic Epilepsy panel saw larger increases in the number of diagnostic genes and P/LP variants than the EOSE panel, both panels saw similar proportional increases in the number of diagnostic P/LP variants (148 vs. 133%). These findings indicate that patients tested with both broad panels would benefit from a shorter re-analysis frequency. However, studying the ways changes accumulate in each panel on a month-by-month basis reveals a more nuanced pattern.

While the PanelApp Australia Genetic Epilepsy panel is updated on a monthly basis, the Genomics England PanelApp EOSE panel is updated annually. These differences have implications for re-analysis—namely that the EOSE sees infrequent large changes and re-analysis should potentially be coupled with scheduled panel updates, although interim analysis will still benefit from changes in P/LP variants alone. Conversely, for healthcare systems and diagnostic laboratories that opt for panel updates at shorter intervals, patients may benefit from corresponding shorter periods between re-analysis. This more agile approach to updates potentially facilitates faster diagnosis through both primary analysis and re-analysis though may have significant implications for laboratory workflows.

Analysis of the Specific panels revealed a significant finding. Each of the three Specific panels showed that the number of P/LP variants still increased, even when the number of available diagnostic genes did not. These findings demonstrate that patients tested with these Specific panels would still benefit from re-analysis as would patients tested using traditional amplicon-based assays. While the field is moving away from amplicon-based assays, a significant amount of stored data from these tests is still available. However, given the large amount of gene content change^21^, there is still a significant benefit to re-sequencing patients previously tested using older assays and employing a virtual gene panel approach on an exome/genome backbone.

We acknowledge that the re-analysis of genomic information is laborious, time consuming and currently places significant workload on laboratories and clinicians. However, an approach that enables re-analysis based on a shorter period of time or is triggered by the amount of information newly available to laboratories will provide more epilepsy patients with an earlier diagnosis and will likely provide healthcare system benefit. The value of rapid, timely diagnosis in epilepsy, especially infantile epilepsy, are well recognised^33,34^, and any approach that has the potential to enable access to precision treatments, prevent additional tests, and ineffective or unnecessary treatments is worth exploring. Moreover, if a panel is associated with a lower rate of change, delaying re-analysis until the amount of new information passes a pre-defined threshold could prevent an additional expense for the health system when the likelihood of identifying a diagnostic variant remains low. While re-analysis has the potential to help health services realise the quadruple aim of healthcare (improved patient experience, improved clinician/provider experience, improved population health, sustainable cost) any provider looking to adopt re-analysis enabled care must ensure that there is sufficient resources, systems and policies to support this.

This work shows the importance of knowledge resources about gene- and variant-disease associations to the analysis and re-analysis of genomic data, and the need for clinicians, laboratory scientists and researchers involved in genomics to continuously contribute to their growth and maintenance^25,26,28^. Health services can also support re-analysis by developing policies to ensure that data are routinely shared with resources like ClinVar. National infrastructure to support knowledge and data sharing such as PanelApp and Shariant^35^ have been developed to address this and decrease the barriers to individual laboratories contributing.

The results from this comparison also highlight the distinct benefits to the different types of panel management employed by PanelApp Australia and the Genomics England PanelApp. One benefit of the more frequent approach employed by PanelApp Australia was highlighted by the ultra-rapid review of the diagnostic genes that were found to be unique to the Genomics England EOSE panel. Within twelve hours of being made aware of that there were diagnostic grade genes that were not present on the Genetic Epilepsy panel, each gene had been reviewed, and those with appropriate amounts of evidence were added to the Australian panel.

For patients, clinicians and laboratories to benefit from the accumulation of new knowledge, new systems to automatically incorporate new knowledge about genes and variants and interrogate existing data need to be developed and evaluated. Resources like PanelApp and ClinVar could help enable differential timing of re-analysis if the changes in data were readily accessible through visualisations. Figures that are automatically updated that display how the number of P/LP variants or diagnostic genes have changed over time, would allow laboratories storing data from epilepsy patients to identify potentially clinically significant bursts at a glance. These changes would benefit all patients needing re-analysis, not just those tested with an epilepsy panel.

We acknowledge that epilepsy is a heterogenous disease and is associated with a broad range of clinical phenotypes. As epilepsy patients are often tested with large, ‘broad’ epilepsy panels, with a patient’s phenotypic information being used during curation to focus on more specific genes^36^, we studied the same molecular tests offered to these patients. However, as our understandings of the specific molecular components of epilepsy improve, future studies should look to collect additional information to examine the role that additional phenotypic information could play in re-analysis.

## Conclusion

Through the application of Vigelint, it was possible to characterise how our understanding of the molecular components of epilepsy are evolving at an unprecedented resolution. Analysing the changes in the number of pathogenic variants in clinically significant epilepsy genes over a period of five years, allowed us to determine the average rate of change, as well as the identify sudden bursts of new, clinically relevant information. Together, these results provide evidence that can guide how the genomic information for epilepsy patients who underwent testing but did not receive a molecular diagnosis can be re-analysed. Moreover, it is our hope that by sharing Vigelint with other researchers, this approach will mature and be used to characterise how our understanding of other conditions is evolving.

### Supplementary Information

Supplementary Tables.

## Data Availability

All data analyzed here can be downloaded from PanelApp, PanelApp Australia and ClinVar. Results from the analysis of this data for the current study are available from the corresponding author on reasonable request.
